# Coverage, inequity and predictors of hepatitis B birth vaccination in Myanmar from 2011–2016: results from a national survey

**DOI:** 10.1186/s12913-022-07902-w

**Published:** 2022-04-19

**Authors:** August C. T. Anderson, Adam Richards, Kevin Delucchi, Mandana Khalili

**Affiliations:** 1 School of Medicine, University of California, San Francisco, 505 Parnassus Avenue, San Francisco, CA 94143 USA; 2 Department of Global Health, Milken Institute School of Public Health, The George Washington University, 950 New Hampshire Ave, Washington, DC, NW 20052 USA; 3 Department of Psychiatry and Behavioral Sciences, University of California, San Francisco, 401 Parnassus Ave, San Francisco, CA 94143 USA; 4 Department of Medicine, University of California, San Francisco, Zuckerberg San Francisco General Hospital, 1001 Potrero Ave, Building 5, Suite 3D4, San Francisco, CA 94110 USA

**Keywords:** Hepatitis B / prevention & control, Hepatitis B Vaccines / therapeutic use, Infectious Disease Transmission, Vertical / prevention & control, Maternal-Child Health Services, Quality of Health Care

## Abstract

**Background:**

Hepatitis B virus birth dose (HepB-BD) vaccination is recommended to reduce mother to infant transmission. We evaluated the HepB-BD status of women who gave birth between 2011 and 2016 (*N* = 3,583) using the 2015–2016 Myanmar Demographic and Health Survey.

**Methods:**

Frequency distributions of HepB-BD vaccination across maternal and health system factors, concentration indices, and logistic regression models were used to estimate coverage, inequity, and factors associated with vaccination.

**Results:**

The majority of participants were younger than 30 years of age, lived in rural areas, and were multiparous. Almost all received antenatal care (ANC), but only 43% received recommended ANC services, and 60% gave birth at home. The overall HepB-BD coverage rate was 26%. Vaccination coverage was higher in urban areas and was inequitably concentrated among children of more educated and wealthier women. HepB-BD coverage was also positively associated with receipt of ANC at non-governmental facilities, and delivery at a facility, skilled provider at birth and Cesarean delivery. After adjusting for sociodemographic and health system factors, receipt of the HepB-BD was positively associated with weekly media exposure, receipt of recommended ANC, and Cesarean delivery, and inversely associated with home delivery.

**Conclusions:**

Both socioeconomic and health systems factors influenced suboptimal and inequitable vaccination coverage. Improved access to quality ANC and delivery services may increase HepB-BD coverage although targeted approaches to reach home births are likely required to achieve national goals.

**Supplementary Information:**

The online version contains supplementary material available at 10.1186/s12913-022-07902-w.

## Background

An estimated 257 million people worldwide live with chronic Hepatitis B virus (HBV) infection, predominantly in Asia [1]. Untreated chronic HBV can progress to liver cirrhosis, liver failure, and hepatocellular carcinoma [2]. Most individuals with chronic HBV acquire the infection prior to 5 years of age, and the most common route of transmission in endemic areas is perinatal vertical transmission with approximately 90% of infants born to HBV-infected mothers developing chronic infection [1]. In 2016, the World Health Organization (WHO) set a goal for the elimination of viral hepatitis by 2030, including a 90% reduction in mother-to-child transmission of Hepatitis B [1]. To meet this target, the WHO called for provision of a monovalent birth dose (HepB-BD) vaccine within 24 h of delivery but in 2019 the global coverage with the HepB-BD was only 43% [1, 3]. Although it is theoretically possible to eliminate nearly all vertically transmitted cases of HBV by augmenting the HepB-BD with additional interventions (antenatal antiviral treatment and newborn receipt of hepatitis B immunoglobulin) targeted to pregnant women with high viral loads [4], timely HepB-BD vaccination alone reduces vertical transmission risk by approximately 68% overall [5], and by approximately 96% for women who are hepatitis B e antigen-negative [6].

In Southeast Asia the overall prevalence of HBV among children below the age of 5 has been reported at 1.1% [7], and the country with the highest estimated prevalence (3.8%) is Myanmar [8]. Myanmar is also estimated to have the highest rate of perinatal chronic infections in the region, at 16 per 1000 live births [8]. Although Myanmar has the lowest Human Development Index score in Southeast Asia [9], the country aspires to provide Universal Health Coverage (UHC) for the population [10]. The Myanmar National Hepatitis Control Program has prioritized expansion of the HepB-BD vaccination program while focusing on areas of greatest need in a manner that is explicitly pro-poor [10]. According to the WHO, national coverage with the HepB-BD was 1% in 2017 and 17% in 2019 [11], but population-based estimates of coverage, inequity, or maternal and health system factors associated with vaccination are not known.

Our study aimed to investigate national coverage and inequity of the HepB-BD and to examine the relationship between maternal and health system factors and HepB-BD vaccination. Our a priori hypothesis was that accounting for utilization of antenatal and delivery care services would attenuate many of the expected associations between socio-demographic factors and HepB-BD coverage.

## Methods

### Data source and study population

We used the first Myanmar Demographic and Health Survey (DHS) [12], a cross-sectional nationally-representative survey conducted by the Myanmar Ministry of Health & Sports (MOHS) between December 2015 and July 2016. The survey followed a stratified two-stage sample design based on the 2014 census, and was weighted to allow representative estimates for urban and rural areas as well as for each of the seven States and eight Regions of Myanmar. The MOHS interviewed 12,885 women age 15–49 years old from 12,500 households and responses from women who delivered between 2011 and 2016 were analyzed. For this study, we utilized the structured Woman’s Questionnaire developed for the global DHS program and modified for the Myanmar survey, which included questions on maternal demographic and socioeconomic characteristics as well as antenatal and delivery healthcare services.

### Assessment of HepB-BD vaccination

HepB-BD vaccination was defined as receipt of the HepB-BD within 24 h of delivery. Participants’ children were coded as vaccinated within 24 h based on documentation in the child’s health card or maternal report. Children were coded as not vaccinated if the health card did not document the HepB-BD vaccine or participants denied receipt or if participants didn’t know their child’s vaccination status. In a *post-hoc* sensitivity analysis we limited the definition of vaccination to the children who had the vaccine documented on their health card (185 participants, 5%). The vaccination status of the most recent child was analyzed. For multiple gestation deliveries, the status of a single child was assessed, as all twins and triplets either had missing (< 1%) or concordant data.

### Conceptual model

Our selection of potential predictors of HepB-BD vaccination and development of analytical models drew on the combination of two conceptual frameworks. Andersen’s model of medical care utilization emphasizes the complementary contributions of predisposing factors (such as demographics and social structure) and enabling factors (such as the availability of medical care) [13]; Nutting’s model of health system performance highlights the sequential nature of health service utilization [14]. A medical care utilization model is appropriate to conceptualize access to vaccines given at birth that unlike most other Expanded Program of Immunization (EPI) vaccines can be administered by clinical staff who provide obstetric and neonatal services, and as such may be closely correlated with other processes indicative of an accessible and well-functioning clinical care system. Similar to many low-income countries, in Myanmar the monovalent HBV vaccine is not administered by EPI outreach staff; although MOHS policy permits midwives to administer vaccines to newborns at home, the MOHS has not procured monovalent vaccine doses for this purpose [15].

A simplified two-step pathway can be used to describe HepB-BD vaccination in Myanmar: first, the mother must deliver within a clinical care system where the vaccine is available; second, the health system must administer the vaccine within 24 h after birth. Socioeconomic inequities in access to ANC and delivery care have been documented in Myanmar [16]; inequities in treatment are less well understood but are plausible due to factors such as out-of-pocket costs [17], or discrimination in healthcare settings [10].

### Predictor variables

Potential predictors were selected based on their prior associations with receipt of the HepB-BD [18–24] or other childhood vaccines in Myanmar [25] or their theoretical associations with receipt of the HepB-BD based on conceptual frameworks. Continuous variables were categorized a priori. Maternal and infant characteristics included maternal age at delivery, parity, maternal education, urban residence (vs rural), geographic areas categorized in four zones: Delta (Ayeyarwaddy, Bago, Yangon), Coastal (Mon, Rahkine, Taninthayi), Central (Magway, Mandalay, Naypyitaw, Sagaing), and Hilly (Chin, Kachin, Kayah, Kayin, Shan), household wealth index quintile (calculated by the DHS using principal component analysis of household assets, building materials, and sanitation) [26], and low infant birthweight (< 2,500 g). Health system variables were organized into domains related to antenatal- (ANC), delivery- and postnatal care. Recommended ANC services was defined as the combination of 3 tests (blood pressure, urine, and blood) with care from a skilled provider (physician, nurse, midwife, or lady health visitor), and modeled as a binary variable versus less or no ANC. Prepartum tetanus vaccination was included as an complementary indicator of ANC quality. Among participants who received any ANC, early utilization of ANC (attending the first ANC contact during the first trimester), the number of ANC contacts, and ANC location (government hospital only, government clinic only, private facility only [private hospitals and clinics, Myanmar or international non-governmental organizations, or other locations], home only, or multiple locations) were also evaluated. Delivery care predictors included delivery by a skilled provider, delivery location and mode of delivery; postnatal care was indicated by receipt of a postnatal health check within 24 h by a skilled provider. Additional predictors included year of delivery and weekly media exposure (use of either print, radio or TV media at least once per week, vs less often).

Survey responses of ‘don’t know’ (early utilization of ANC, number of ANC contacts, prepartum tetanus vaccination, birthweight, postnatal health check) and ‘unmeasured’ (birthweight), were combined with missing values and labeled as a separate category in the analysis accounting for 37% of predictor variables. The percent of missing data for each predictor was as follows: 2% of prepartum tetanus vaccination and mode of delivery, 5% of location of ANC, 6% of early ANC and number of ANC contacts, 24% of postnatal health check, 50% of low birthweight. In a sensitivity analysis we used multiple imputation with chained equations to create 50 datasets with imputed values for all variables, and no substantial differences in the estimated effect sizes were found when compared to the multivariable model without multiple imputation (data not shown).

### Statistical analysis

Participant and health system characteristics were described with frequency distributions and differences between groups were evaluated with the Rao‐Scott χ^2^ test. The health concentration index [27] was calculated to evaluate inequality of vaccination rates ranked by household wealth and maternal education (described in Appendix 1). Two logistic regression models were used to assess factors associated with HepB-BD vaccination. First, a model was fit using predisposing and enabling factors (geographic region, delivery year, and maternal characteristics) to assess demographic and socioeconomic patterns of HepB-BD vaccination (Model 1). Second, we added to the model health system factors, as well as additional predictors that had significant (p ≤ 0.2) univariate associations with the outcome (parity, media exposure, Cesarean delivery, postnatal health check). We then used Allen-Cady modified backwards selection (Wald test, *p* < 0.1), resulting in the removal of parity and the postnatal health check from the final model (Model 2). Colinearity was evaluated using weighted linear regression, and the Variance Inflation Factor (VIF) was < 3 for all predictors, signifying low concern for collinearity, while a link test and the Archer–Lemeshow Goodness-of-Fit test [28] indicated no evidence for specification errors, missing interactions or nonlinear variables. Odds Ratios (OR) and their 95% Confidence Intervals (CI) were used to estimate the association of factors with the outcome; predicted probabilities were calculated using average marginal effects at representative values of maternal and health system characteristics. Data were analyzed using survey features that incorporated the complex sample design and survey weights. All *p*-values were two-sided and *p* < 0.05 was considered statistically significant. All analyses were performed with Stata 16.0 (StataCorp, College Station, TX, USA), using the conindex program to calculate concentration indices [29].

### Ethical approval

The present study was considered exempt by the UCSF Institutional Review Board. The survey protocol was reviewed and approved by the Ethics Review Committee on Medical Research including Human Subjects in the Department of Medical Research of the Myanmar Ministry of Health and Sports.

## Results

### Participant and health system characteristics

**Fig. 1:**
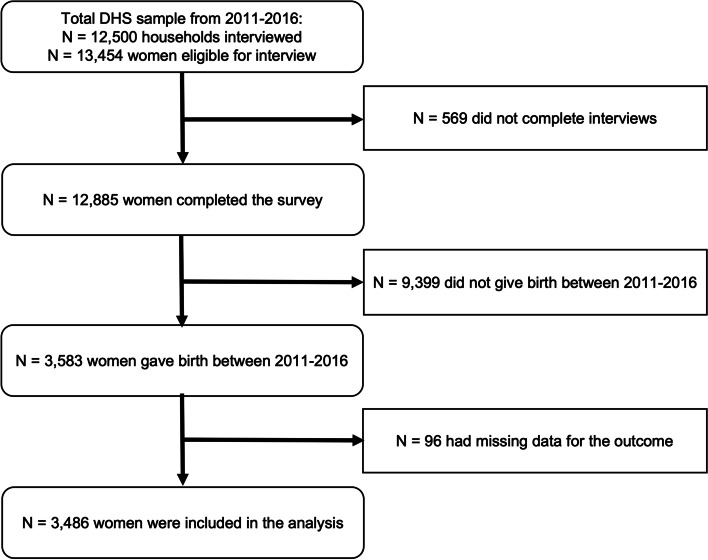
Participant flow

Of the 12,885 women who completed the DHS Women’s Questionnaire, we excluded 9,399 who did not give birth between 2011 and 2016, and 96 who had missing data for HepB-BD status (see Fig. 1). Vaccination rates were compared between women with complete versus missing data for each predictor, and participants with missing data for ANC location, early ANC, number of ANC contacts, mode of delivery, low birthweight, or postnatal health check all had significantly lower HepB-BD coverage rates versus participants with non-missing data (data not shown).

The participant characteristics and health system factors stratified by their child’s vaccination status are summarized in Table 1. Most participants were younger than 30 years of age at the time of delivery (62%), multiparous (66%), completed at least a primary school education (84%), reported at least weekly media exposure (60%), and lived in rural areas (76%) or the Delta and Central zones of Myanmar (66%) (see Table A1 for specific regions and states). Almost all women had at least 1 ANC contact (92%), but fewer than half of these received recommended ANC services from a skilled provider (43%), initiated ANC during their first trimester (46%), or reported ≥ 8 ANC contacts. In contrast, most women received ANC exclusively at government facilities and a majority of women had a skilled delivery provider (64%) or gave birth at home (59%). One third (32%) of infants had a postnatal health visit within 24 h by a skilled provider.

**Table 1:** Maternal, health system and infant characteristics stratified by HepB-BD vaccine status

Characteristics	Participants *N* = 3486	Did not receive BD vaccine *n* = 2587	Received BD vaccine *n* = 899
Year of delivery*			
2011	422 (12)	297 (11)	126 (14)
2012	647 (19)	489 (19)	158 (18)
2013	658 (19)	464 (18)	193 (22)
2014	791 (23)	554 (21)	237 (26)
2015	858 (25)	683 (26)	175 (19)
2016	110 (3)	100 (4)	11 (1)
**Maternal characteristics**			
**Age at delivery**			
15–20	369 (11)	293 (11)	76 (8)
21–25	843 (24)	620 (24)	223 (25)
26–30	946 (27)	687 (27)	259 (29)
30–49	1329 (38)	987 (38)	342 (38)
Primiparous*	1202 (34)	817 (32)	386 (43)
Education*			
None	558 (16)	463 (18)	95 (11)
Primary	1790 (51)	1406 (54)	384 (43)
Secondary	844 (24)	563 (22)	281 (31)
Higher	294 (8)	155 (6)	139 (15)
Weekly media exposure*	2103 (60)	1434 (55)	670 (74)
Household wealth quintiles*			
Poorest	918 (26)	738 (29)	181 (20)
Poorer	776 (22)	612 (24)	164 (18)
Middle	654 (19)	474 (18)	180 (20)
Richer	577 (17)	399 (15)	178 (20)
Richest	560 (16)	364 (14)	197 (22)
Urban area*	821 (24)	454 (18)	366 (41)
Geographic zone*^a^			
Delta	1176 (34)	793 (31)	383 (43)
Central	1105 (32)	859 (33)	246 (27)
Coastal	454 (13)	377 (15)	77 (9)
Hilly	750 (22)	558 (22)	192 (21)
**Health system characteristics**			
Recommended ANC services*^b^	1509 (43)	926 (36)	583 (65)
**First trimester ANC** (*n* = 3033)	1398 (46)	1004 (46)	395 (46)
**Prepartum tetanus vaccine** (*n* = 3429)	2938 (93)	2141 (93)	798 (93)
Number of ANC contacts* (*n* = 3286)			
0	261 (8)	236 (10)	25 (3)
1–3	968 (29)	789 (33)	179 (21)
4–7	1447 (44)	1049 (43)	398 (46)
≥ 8	610 (19)	341 (14)	269 (31)
ANC location* (*n* = 3308)			
Government hospital only	648 (21)	389 (18)	259 (3)
Government clinic only	1345 (44)	1062 (48)	283 (33)
Private hospital/clinic only	284 (9)	149 (7)	135 (16)
Home only	552 (18)	456 (21)	96 (11)
Multiple locations	866 (7)	525 (24)	341 (40)
Delivery location*			
Government hospital	1078 (31)	598 (23)	479 (53)
Government clinic	100 (3)	76 (3)	24 (3)
Private hospital/clinic	258 (7)	114 (4)	144 (16)
Home	2050 (59)	1798 (70)	252 (28)
Skilled delivery provider*	2233 (64)	1473 (57)	760 (85)
Cesarean delivery* (*n* = 3474)	686 (20)	333 (13)	353 (40)
Postnatal health check* (*n* = 2663)	865 (32)	583 (31)	281 (37)
**Infant characteristics**			
**Low birthweight** (*n* = 1732)	132 (8)	91 (9)	41 (6)

*n* are weighted counts, rounded to the nearest whole number

^*^p < 0.05

^a^Geographic zones: Delta (Ayeyarwady, Bago, Yangon), Central (Magway, Mandalay, Naypyitaw, Sagaing), Coastal (Mon, Rakhine, Tanintharyi), Hilly (Chin, Kachin, Kayah, Kayin, Shan)

^b^Recommended ANC services included blood pressure, urine and blood tests from a skilled provider

### Coverage and inequity of HepB-BD vaccination

Among the 3,486 women who gave birth between 2011 and 2016, children of 899 women (26%) received the HepB-BD vaccine, based on documentation in the child’s health card (5%) or maternal report (21%).

The Wagstaff-normalized concentration index (*C*) provides a summary measure of health inequality that takes into account the entire distribution of vaccination coverage across values of ordinal (ranked) variables, and was positive and large for both years of maternal education (*C* = 0.25, standard error [SE] 0.03) and household wealth index values (*C* = 0.17, SE 0.03). This indicates a skewed pattern of vaccination, with lower rates of vaccination among children born to women with fewer years of education, and those born into poor households.

**Fig. 2:**
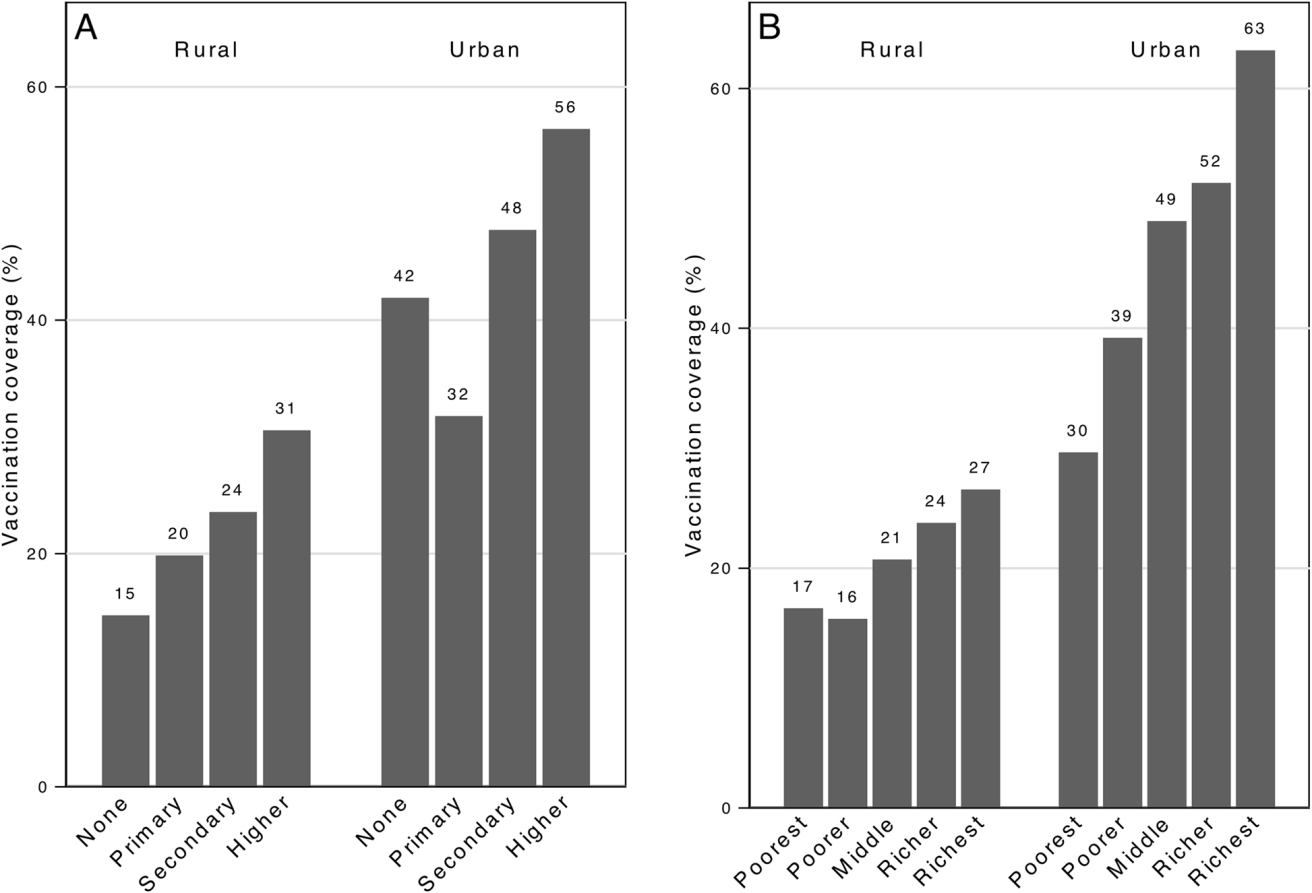
Hepatitis B birth dose vaccination coverage (%) by (**A**) maternal education level and (**B**) household wealth index quintiles, stratified by urban and rural residence

Table 2 reports receipt of the HepB-BD by maternal and healthcare service characteristics. In unadjusted analyses, vaccination rates were higher among newborns delivered by women who were older, primiparous, had weekly media exposure, and who lived in the Delta zone (see Table A1 for each specific Region and State). Large, graded socioeconomic inequities in vaccination coverage were observed according to maternal educational attainment and household wealth, in both rural and urban settings. Vaccination coverage among newborns delivered by women with the highest level of education or household wealth quintile in urban areas (56% and 63%, respectively) was over three times the coverage observed among children born to women with no education or from the poorest wealth quintile in rural areas (15% and 17%, Fig. 2).

**Table 2:** Receipt of BD vaccination by maternal, health system and infant characteristics

**Characteristics**	**Received** **BD vaccine**	**Univariate Associations**		**Model 1**^c^ ***N* = 3486**		**Model 2**^d^ ***N* = 3486**	
	***n*(row %)**	**OR**	**95% CI**	**aOR**	**95% CI**	**aOR**	**95% CI**
**Delivery year**							
2011	126 (30)	1.0 (ref)		1.0 (ref)		1.0 (ref)	
2012	158 (24)	0.76	0.56, 1.04	0.75	0.55, 1.02	0.72	0.51, 1.02
2013	193 (29)	0.99	0.74, 1.32	1.00	0.74, 1.34	0.85	0.61, 1.19
2014	237 (30)	1.01	0.75, 1.37	0.96	0.71, 1.31	0.72	0.52, 0.99
2015	175 (20)	0.60*	0.45, 0.81	0.56*	0.41, 0.77	0.41*	0.29, 0.58
2016	11 (10)	0.25*	0.09, 0.66	0.25*	0.10, 0.62	0.18*	0.07, 0.44
**Maternal characteristics**							
**Age at delivery**							
15–20	76 (21)	1.0 (ref)		1.0 (ref)		1.0 (ref)	
21–25	223 (26)	1.40	0.99, 1.97	1.35	0.94, 1.94	1.20	0.83, 1.73
26–30	259 (27)	1.46*	1.02, 2.09	1.41	0.97, 2.03	1.26	0.86, 1.86
31–49	342 (26)	1.34	0.98, 1.84	1.31	0.94, 1.82	1.09	0.78, 1.53
**Primiparous**							
No	513 (22)	1.0 (ref)					
Yes	386 (32)	1.63*	1.34, 1.98				
**Education**							
None	95 (17)	1.0 (ref)		1.0 (ref)		1.0 (ref)	
Primary	384 (21)	1.33	0.95, 1.87	1.12	0.79, 1.59	0.78	0.55, 1.11
Secondary	281 (33)	2.43*	1.69, 3.50	1.42	0.98, 2.06	0.76	0.51, 1.14
Higher	139 (47)	4.37*	2.78, 6.85	1.74*	1.06, 2.86	0.80	0.48, 1.33
**Household wealth quintiles**							
Poorest	181 (20)	1.0 (ref)		1.0 (ref)		1.0 (ref)	
Poorer	164 (21)	1.09	0.83, 1.44	1.02	0.79, 1.59	0.81	0.59, 1.12
Middle	180 (27)	1.55*	1.16, 2.07	1.51*	1.11, 2.06	1.00	0.71, 1.41
Richer	178 (31)	1.82*	1.30, 2.54	1.68*	1.18, 2.38	1.97	0.67, 1.42
Richest	197 (35)	2.21*	1.55, 3.16	1.96*	1.31, 2.94	0.90	0.58, 1.41
**Urban area**							
No	532 (20)	1.0 (ref)		1.0 (ref)		1.0 (ref)	
Yes	366 (45)	3.23*	2.46, 4.24	2.68*	2.05, 3.50	1.13	0.83, 1.55
**Geographic zone**^a^							
Delta	383 (33)	1.0 (ref)		1.0 (ref)		1.0 (ref)	
Central	246 (22)	0.59*	0.44, 0.80	0.62*	0.45, 0.83	0.65*	0.48, 0.88
Coastal	77 (17)	0.43*	0.30, 0.61	0.56*	0.40, 0.80	0.61*	0.42, 0.89
Hilly	192 (26)	0.71	0.51, 1.00	0.81	0.58, 1.13	0.99	0.67, 1.46
**Weekly media exposure**							
No	229 (17)	1.0 (ref)				1.0 (ref)	
Yes	670 (32)	2.35*	1.88, 2.93			1.36*	1.07, 1.72
**Health system characteristics**							
**Recommended ANC services**^b^							
No	291 (17)	1.0 (ref)				1.0 (ref)	
Yes	583 (39)	3.31*	2.66, 4.12			1.65*	1.24, 2.19
**Prepartum tetanus vaccine** (*n* = 3429)							
No	58 (25)	1.0 (ref)					
Yes	798 (27)	1.11	0.77, 1.59				
** First trimester ANC** (*n* = 3429)							
No	457 (28)	1.0 (ref)				1.0 (ref)	
Yes	395 (28)	1.01	0.81, 1.26			0.91	0.72, 1.15
**Number of ANC contacts** (*n* = 3289)							
0	25 (10)	0.47*	0.26, 0.84			0.70	0.37, 1.32
1–3	179 (19)	1.0 (ref)				1.0 (ref)	
4–7	398 (28)	1.67*	1.32, 2.11			1.02	0.78, 1.33
≥ 8	269 (44)	3.48*	2.60, 4.65			1.23	0.87, 1.75
**ANC location** (*n* = 3308)							
Government hospital only	259 (40)	1.0 (ref)				1.0 (ref)	
Government clinic only	283 (21)	0.40*	0.31, 0.51			0.80	0.59, 1.08
Private hospital/clinic only	135 (48)	1.36	0.95, 1.94			0.98	0.62, 1.56
Home only	96 (17)	0.31	0.22, 0.44			1.01	0.68, 1.50
Multiple locations	81 (37)	0.89*	0.60, 1.34			0.89	0.58, 1.37
**Delivery location**							
Government hospital	479 (44)	1.0 (ref)				1.0 (ref)	
Government clinic	24 (24)	0.40	0.21, 0.76			0.50*	0.26, 0.96
Private hospital/clinic	144 (56)	1.57*	1.09, 2.25			1.19	0.77, 1.84
Home	252 (12)	0.17*	0.14, 0.22			0.32*	0.23, 0.44
**Skilled delivery provider**							
No	139 (11)	1.0 (ref)				1.0 (ref)	
Yes	760 (34)	4.15*	3.15, 5.46			1.11	0.77, 1.62
**Cesarean delivery** (*n* = 3474)							
No	538 (19)	1.0 (ref)				1.0 (ref)	
Yes	353 (51)	4.42*	3.59, 5.45			1.49*	1.14, 1.95
**Postnatal health check** (*n* = 2663)							
No	476 (26)	1.0 (ref)					
Yes	281 (33)	1.34*	1.06, 1.68				
**Infant characteristics**							
**Low birthweight** (*n* = 1732)							
No	615 (38)	1.0 (ref)				1.0 (ref)	
Yes	41 (31)	0.72	0.48, 1.09			0.75	0.48, 1.15

*n *are weighted counts, rounded to the nearest whole number. *OR* Odds Ratio, *aOR* Adjusted Odds Ratio, *CI* Confidence Interval, rounded to the nearest hundredth, *Ref* reference value

^*^ *p* < 0.05

^a^Geographic zones: Delta (Ayeyarwady, Bago, Yangon), Central (Magway, Mandalay, Naypyitaw, Sagaing), Coastal (Mon, Rakhine, Tanintharyi), Hilly (Chin, Kachin, Kayah, Kayin, Shan)

^b^Recommended ANC services included blood pressure, urine and blood tests from a skilled provider

^c^Model 1 includes maternal sociodemographic characteristics (delivery year, age, education, household wealth, geographic zones, urban area)

^d^Model 2 is adjusted for all variables in Model 1 as well as media, health system, and infant factors

HepB-BD vaccination rates were associated with several ANC and delivery factors, including the receipt of recommended ANC services or delivery care from a skilled provider, the number of ANC contacts, and the location of both ANC and delivery services (Table 2). Newborns of women who received ANC or delivery care at a government hospital or a private facility had higher rates of vaccination; newborns had lower rates of vaccination if ANC or delivery care occurred at a government clinic or at home. Delivery by Cesarean-section, and receipt of a postnatal health check also were associated with receipt of the HepB-BD. Vaccination rates were lowest for women who did not have any ANC (10%), or who delivered at home (12%) or without a skilled provider (11%). Only deliveries in a private facility or via Cesarean-section were associated with vaccination rates above 50%.

Table 2 also shows results of the multivariable analyses evaluating independent associations with HepB-BD vaccination. Our first model (Model 1) adjusted for delivery year, geographic region and maternal sociodemographic factors in order to describe the patterning of HepB-BD vaccination prior to accounting for other correlated health services. Large and independent associations with the HepB-BD were found for maternal education, household wealth, and urban residence in this partially adjusted model. In our model that further adjusted for media exposure and health system factors (Model 2), the HepB-BD was no longer independently associated with the sociodemographic factors in Model 1. Children born to women with weekly media exposure were more likely to receive the HepB-BD vaccine, compared to those with less frequent exposure (aOR 1.36, 95% CI 1.07, 1.72). Receipt of recommended ANC services from a skilled provider increased the likelihood of vaccination (aOR 1.65, 95% CI 1.24, 2.19) compared to any or no ANC. The location of delivery had the largest effect on the likelihood of vaccination. Compared to children born in a government hospital, the odds of vaccination was lower for children delivered in government clinics (aOR 0.50, 95% CI 0.26, 0.96) and substantially lower for children born at home (aOR 0.32, 95% CI 0.23, 0.44). Delivery via Cesarean-section was also associated with HepB-BD vaccination (aOR 1.49, 95% CI 1.14, 1.95). Under an ideal counterfactual scenario in which all pregnant women in Myanmar were exposed to weekly media, received early ANC and recommended ANC services from a skilled provider, completed at least 8 ANC contacts at a private facility, and delivered in a private facility, the predicted probability of HepB-BD vaccination would have been 0.49, compared to 0.10 if all women did not have weekly media exposure, had no ANC and delivered at home without a skilled delivery provider.

## Discussion

Using data from a nationally-representative survey we estimate that 26% of newborns born in Myanmar between 2011 and 2016 received the monovalent hepatitis B birth dose vaccine within 24 h after birth. Our estimate is higher than the official HepB-BD coverage rate reported by the WHO (1% in 2017 and 17% in 2019) [3]. While the reasons for this discrepancy cannot be addressed directly from our study, several factors may have contributed. First, official estimates based on administrative reports may reflect biased estimates of both the numerator (e.g. inaccurate counting or reporting, particularly from births in private facilities) and denominator (e.g. outdated population estimates) [30–32]. Second, our reliance on parental report for a large proportion (79%) of vaccination events may have led to over-estimation of HepB-BD coverage in our study. In a *post-hoc* analysis limited to participants whose receipt of the HepB-BD vaccine was documented on a health card and verified by the interviewer (excluding parental report), the national HepB-BD coverage was estimated to be 5% (Appendix 2).

Our estimated coverage fell far short of Myanmar’s goal for 2020 (80% coverage) and the WHO goal of 90% by 2030 [1, 33]. Myanmar previously provided the HepB-BD vaccine between 2003 and 2009 with support from Gavi, the Vaccine Alliance [15], and reportedly achieved 33% coverage throughout the country in 2003 [11]. However after Gavi ended its support in 2009, the Myanmar MOHS only administered the vaccine at large hospitals [34], and the national coverage for hospital-based births was reportedly 20–25% between 2010 and 2014 [35]. Although our study suggests that coverage in government hospitals was higher during the survey period (44% from 2011 to 2016), it remained far below the national target for hospital-based births in 2017 (75%) [33].

We observed large socioeconomic gradients in HepB-BD vaccination rates similar to those observed for other clinical and public health services in Myanmar, including childhood vaccination [16, 25, 36, 37]. HepB-BD vaccination rates were almost four times higher among children born into the wealthiest households in urban areas, compared to children born into the poorest rural households (63% vs 17%). Education, wealth and urbanicity retained their positive, independent associations with vaccination in our partially adjusted model (Model 1).

In our full model (Model 2), we found that the associations of socioeconomic factors and HepB-BD vaccination were entirely accounted for by weekly media exposure and health system factors. At least three phenomena likely contributed to the larger than expected attenuation of socioeconomic inequities in HepB-BD coverage. First, the magnitude of the inequities in utilization of ANC and delivery care services were larger than anticipated. In *post-hoc* analyses we calculated concentration indices for all factors that retained statistically significant associations with vaccination in our final model except geographic zone (weekly media exposure, recommended ANC care, facility delivery and Cesarean delivery), and found that they demonstrated large and inequitable distributions (*C* between 0.22 and 0.46) according to both household wealth and maternal education.

Second, the correlations between HepB-BD vaccination and other ANC and delivery factors was stronger than anticipated. For example, all Cesarean surgeries were conducted by a skilled provider and they occurred almost exclusively (98%) in facilities with the highest rates of vaccination (government hospitals or private hospitals and clinics, data not shown). The high correlation of the HepB-BD with other clinical services is consistent with our conceptual model and highlights how HepB-BD vaccines are unique among recommended immunizations in Myanmar in that they are administered almost exclusively by clinicians in healthcare settings. The greater separation between clinical services and vaccination by EPI teams likely partially accounts for the contrast between our results and those of a study in Myanmar of childhood vaccination, that found wealth-related inequalities in complete vaccine coverage persisted after controlling for ANC contacts [25].

The third likely contributor to the large attenuation of inequities in HepB-BD vaccination coverage after adjustment for clinical factors is that the likelihood of receiving the vaccine is in fact similar for individuals who overcome barriers to accessing ANC and delivery services. This unexpected, though promising, finding suggests that additional barriers to the HepB-BD such as low health literacy, discrimination and out-of-pocket fees to purchase vaccines may not substantially impact the likelihood of receiving the HepB-BD for newborns delivered to women who can access healthcare services. Although our findings suggest that greatly enhanced access to ANC and delivery services – for births in facilities and in homes – likely would address inequities in HepB-BD coverage, improved access alone would not be sufficient to achieve coverage targets. Under our counterfactual scenario that assumed all pregnant women received the services associated with HepB-BD vaccination in our full model, the predicted probability of HepB-BD vaccination would have been only 0.49. Home birth (59% of deliveries in our sample) demonstrated the largest (inverse) association with HepB-BD vaccination (aOR 0.32). Achieving high coverage of the HepB-BD vaccine in Myanmar almost certainly will require concerted efforts to redistribute health services, to increase facility deliveries by overcoming barriers to facility delivery [38, 39], to increase availability of the HepB-BD at facilities, and also to develop strategies that facilitate timely administration of the HepB-BD to newborns delivered at home [40, 41].

Subsequent to fielding of the DHS in 2015–2016, public and private actors in Myanmar initiated changes that may have influenced HepB-BD vaccination coverage and modified its associations with the antenatal and delivery care factors presented here. In 2016 the Myanmar MOHS released a national strategic plan for HBV elimination that committed the MOHS to provide the HepB-BD free of charge for facility births and identified strategies for increasing coverage for deliveries that occur in homes or remote geographic locations [15, 33, 35, 42, 43]. For example, the MOHS committed to supply the HepB-BD in remote facilities; it endorsed administration of the HepB-BD in homes by trained birth attendants; and it began to explore temporary storage of vaccines in a controlled temperature chain. The MOHS also embraced universal HBsAg screening of pregnant women, which could be used to identify HBV-infected women to target for interventions to increase facility deliveries or to provide the HepB-BD for home births.

### Limitations

The Demographic and Health Surveys lack a definitive gold standard for vaccination status, such as provider records or serology. Misclassification of our primary outcome is possible, which may have altered the relative associations estimated in our models though it is less likely to have qualitatively influenced our results. Parental report was used to identify most children who received the HepB-BD vaccine (79%) and recall bias may have influenced our estimation of coverage; in a *post-hoc* sensitivity analysis (Appendix 2) the crude and adjusted associations with measured factors were similar for participants whose vaccination status was documented on a health card inspected by the survey interviewer. Although vaccine coverage was lower among children born to women with missing data for predictor variables, we conducted a pre-specified sensitivity analysis using multiply imputed data and obtained similar results (data not shown). The cross-sectional survey design may have resulted in misclassification of sociodemographic factors such as household wealth and location of residence that were assessed at the time of the survey rather than at delivery, but is less likely to have influenced our estimates of the inequity observed for educational attainment, which often remains unchanged after the age when most women give birth. Finally, the DHS did not gather data on additional factors potentially associated with BD vaccination such as ethnicity, religion, HepB-BD-specific knowledge, attitudes and out of pocket costs, or vaccine supply chains; future studies might consider investigating these aspects of HepB-BD vaccination. Nevertheless, the health system factors included in our model were sufficient to explain large socioeconomic inequities in HepB-BD coverage. Finally, our study describes the status of HepB-BD vaccination during a period (2011–2016) when Myanmar did not offer universal free HepB-BD vaccination, which provides a baseline against which to assess future progress towards equitable access to HepB-BD vaccination. Our results may not reflect the subsequent period of public financing for HepB-BD, and we do not capture the adverse impacts of the severe decline in health services access resulting from the military coup in February 2021 [44].

## Conclusion

Overall HepB-BD coverage was suboptimal in Myanmar. Large socioeconomic inequities in HepB-BD vaccination rates were observed, and these inequities were entirely explained by the inequitable distributions of ANC and delivery care factors. Achieving Myanmar’s goal of 80% HepB-BD vaccine coverage by 2030 will likely require targeted efforts to increase the availability of the HepB-BD for facility-based births, and to ensure the HepB-BD reaches children born at home.

## Supplementary Information

**Additional file 1.**

## Data Availability

The data that support the findings of this study are available from the DHS Program at https://dhsprogram.com/data/. Restrictions apply to the availability of these data, which were used under license for this study. Code used for this project is publicly available at doi: 10.17605/OSF.IO/SRQ42.

## Abbreviations

BD: Birth dose
EPI: Expanded Program on Immunization
UHC: Universal health coverage
MOHS: Ministry of health & sports
VIF: Variance inflation factor
