# Supplementary material for: Coverage, inequity and predictors of hepatitis B birth vaccination in Myanmar from 2011–2016: results from a national survey
Source: BMC Health Serv Res. 2022 Apr 19;22:516. doi: 10.1186/s12913-022-07902-w (PMC9016947; doi:10.1186/s12913-022-07902-w)
Supplement: Supplementary file 1 — Additional file 1. [file 12913_2022_7902_MOESM1_ESM.pdf]

Appendices

Table A1: Birth dose hepatitis B vaccination coverage in States and Regions of Myanmar, in unadjusted and adjusted models

Characteristics	Participants N = 3486	Received BD vaccine n = 899			Model 1§ N = 3486		Model 2¶ N = 3486	
	n (col %)	n	Col %	Row %	OR	95% CI	OR	95% CI
**Region/State** (in descending order of vaccination coverage)								
Kayin	110 (3)	43	5	39	1.16	0.69, 1.97	3.48*	1.88, 6.46
Yangon†	377 (11)	135	15	36	1.0 (ref)		1.0 (ref)	
Bago	323 (9)	111	12	35	0.94	0.58, 1.55	2.49*	1.47, 4.22
Magway	264 (8)	80	9	30	0.79	0.45, 1.37	2.33*	1.28, 4.23
Ayeyarwaddy	477 (14)	137	15	29	0.72	0.44, 1.19	2.49*	1.43, 4.34
Kayah	23 (1)	7	1	29	0.75	0.39, 1.43	1.97*	1.09, 3.57
Mandalay	372 (11)	102	11	27	0.67	0.37, 1.23	1.22	0.66, 2.26
Shan	447 (13)	111	12	25	0.59	0.32, 1.09	1.73	0.86, 3.48
Naypyitaw	81 (2)	18	2	22	0.52*	0.30, 0.91	1.27	0.72, 2.25
Taninthayi	99 (3)	19	2	19	0.41*	0.25, 0.70	1.06	0.59, 1.88
Chin	40 (1)	8	1	19	0.41*	0.22, 0.76	2.15*	1.08, 4.26
Kachin	130 (4)	23	3	18	0.39*	0.21, 0.72	1.01	0.54, 1.89
Mon	119 (3)	19	2	16	0.34*	0.18, 0.62	0.71	0.37, 1.36
Rahkine	236 (7)	40	5	17	0.37*	0.19, 0.71	1.72	0.85, 3.48
Sagaing	389 (11)	46	5	12	0.24*	0.12, 0.47	0.61	0.31, 1.19

Notes: n are weighted counts, rounded to the nearest whole number. OR Odds Ratio, CI Confidence Interval, rounded to the nearest hundredth, Ref reference value.
* = $p < 0.05$
†Yangon is the most developed region in the country.
§Model 1 includes maternal sociodemographic characteristics (delivery year, age, education, household wealth, geographic zones, urban area).
¶Model 2 is adjusted for all variables in Model 1 as well as media, health system, and infant factors.

Appendix 1. Concentration Index.

The concentration index [25] C is defined by the health concentration curve, which plots the cumulative distribution of a health outcome or health service, in this case birth dose (BD) vaccination, against the cumulative percentage of the population ranked according to an ordinal variable such as household wealth index or years of maternal education. If the population has the same value for the outcome regardless of socioeconomic rank, then the concentration curve will be represented by a 45-degree line (the line of equality). If the outcome is more common among people with higher socioeconomic rank, then the curve will lie beneath the line of equality. Although concentration curves reveal the presence of relative inequality, they do provide a quantitative summary of the magnitude of in inequality. The concentration index quantifies health inequalities by calculating twice the area between the curve and the line of equality, and is computed by:

$$C = {}^{2}/_{\mu}\, cov(h, r) \qquad \textit{Equation 1}$$

where h is receipt of the BD vaccine, μ is the prevalence of vaccination in the population, r is the fractional rank of an individual within the bounds of the socioeconomic variable (either ordinal wealth scores or years of maternal education), and *cov* is the covariance between vaccination and the fractional rank. For dichotomous outcomes such as vaccination, a Wagstaff-normalized index (C_W) is calculated by dividing C by (1-μ). C_W is bounded between -1 and 1, taking positive values when the concentration curve lies below the line of equality, signifying higher vaccination rates among individuals ranked higher by the socioeconomic variable, and negative values when the concentration curve lies above the line of equality, signifying higher vaccination rates among individuals with lower socioeconomic ranks. A value of 0 indicates either no inequality, or

balanced proportions of higher vaccination rates among both higher- and lower-ranked individuals.

Appendix 2: Sensitivity analysis using an alternative definition of the outcome, limited to birth dose vaccination recorded on a health card inspected by the survey interviewer.

A total of 185 out of 3,486 children included in the analysis had the HBV birth dose vaccine recorded on their health record, representing a weighted prevalence of BD vaccination of 5.3% (95% CI 4.4, 6.3). Table A2 presents results of unadjusted and adjusted analyses for this alternative outcome. Associations are similar to Table 2 in the main manuscript.

Table A2: Receipt of BD vaccination by maternal and health system characteristics

**Characteristics**<br><br>**N = 3486**	**Received BD vaccine**	**Univariable Regression**		**Model 1§**		**Model 2¶**	
	**row %**	**OR**	**95% CI**	**aOR**	**95% CI**	**aOR**	**95% CI**
**Delivery year**							
2011	3.6%	1.0 (ref)		1.0 (ref)		1.0 (ref)	
2012	2.7%	0.75	0.35, 1.60	0.70	0.32, 1.53	0.76	0.35, 1.67
2013	6.9%	1.97	0.95, 4.10	2.06	0.97, 4.37	2.04	0.94, 4.42
2014	8.4%	2.44*	1.25, 4.75	2.34*	1.20, 4.58	2.13*	1.09, 4.15
2015	4.7%	1.32	0.68, 2.54	1.29	0.65, 2.58	1.22	0.61, 2.45
2016	0.3%	0.08*	0.01, 0.66	0.08*	0.01, 0.70	0.07*	0.01, 0.67
**Maternal characteristics**							
**Age at delivery**							
15-20	3.7%	1.0 (ref)		1.0 (ref)		1.0 (ref)	
21-25	4.7%	1.28	0.58, 2.79	1.34	0.60, 3.00	1.28	0.57, 2.89
26-30	6.2%	1.71	0.80, 3.67	1.78	0.85, 3.74	1.73	0.81, 3.71
31-49	5.5%	1.49	0.73, 3.04	1.61	0.80, 3.24	1.42	0.70, 2.89
**Education**							
None	2.4%	1.0 (ref)		1.0 (ref)		1.0 (ref)	
Primary	4.4%	1.85*	1.02, 3.37	1.54	0.79, 3.02	1.20	0.61, 2.37
Secondary	6.9%	2.99*	1.51, 5.92	1.41	0.66, 3.00	0.93	0.42, 2.06
Higher	11.5%	5.23*	2.48, 11.04	1.68	0.72, 3.89	1.01	0.41, 2.45
**Household wealth quintiles**							
Poorest	2.7%	1.0 (ref)		1.0 (ref)		1.0 (ref)	
Poorer	4.1%	1.57	0.83, 2.96	1.56	0.79, 3.02	1.30	0.66, 2.54
Middle	5.4%	2.10*	1.21, 3.64	2.12*	1.13, 3.94	1.53	0.81, 2.92
Richer	9.8%	3.95*	2.25, 6.93	4.05*	2.19, 7.47	2.76*	1.50, 5.05

Richest	6.5%	2.54*	1.33, 4.82	2.44*	1.23, 4.83	1.39	0.70, 2.74
**Urban area**							
No	3.4%	1.0 (ref)		1.0 (ref)		1.0 (ref)	
Yes	11.6%	3.74*	2.56, 5.45	3.18*	2.15, 4.71	1.90*	1.26, 2.86
**Geographic zone†**							
Delta	7.6%	1.0 (ref)		1.0 (ref)		1.0 (ref)	
Central	3.0%	0.38*	0.23, 0.63	0.40*	0.24, 0.69	0.45*	0.26, 0.78
Coastal	2.0%	0.25*	0.12, 0.50	0.36*	0.18, 0.72	0.37*	0.19, 0.75
Hilly	7.0%	0.91	0.57, 1.43	1.10	0.67, 1.80	1.23*	0.75, 2.01
**Weekly media exposure**							
No	2.7%	1.0 (ref)				1.0 (ref)	
Yes	7.0%	2.75*	1.78, 4.25			1.37	0.85, 2.19
**Health system characteristics**							
**Recommended ANC services‡**							
No	2.7%	1.0 (ref)				1.0 (ref)	
Yes	8.8%	3.51*	2.47, 4.99			1.56*	1.02, 2.36
**First trimester ANC** (n = 176)							
No	5.5%	1.0 (ref)				1.0 (ref)	
Yes	6.1%	1.11	0.78, 1.57			1.18	0.81, 1.73
**Number of ANC contacts** (n = 177)							
0	1.3%	0.40	0.10, 1.59			omitted for colinearity	
1-3	3.2%	1.0 (ref)				1.0 (ref)	
4-7	6.8%	2.23*	1.38, 3.60			1.16	0.69, 1.97
≥8	7.8%	2.57*	1.46, 4.53			0.71	0.38, 1.32
**ANC location** (n = 177)							
Government hospital only	9.6%	1.0 (ref)				1.0 (ref)	
Government clinic only	3.6%	0.35*	0.23, 0.55			0.63*	0.40, 1.00
Private hospital/clinic only	9.3%	0.96	0.56, 1.68			0.59	0.32, 1.08
Home only	3.1%	0.30*	0.15, 0.57			0.88	0.40, 1.90
Multiple locations	10.3%	1.08	0.54, 2.16			1.02	0.50, 2.05
**Delivery location**							
Government hospital	9.3%	1.0 (ref)				1.0 (ref)	
Government clinic	2.5%	0.25*	0.06, 0.95			0.15	0.10, 1.44
Private hospital/clinic	13.1%	1.48	0.95, 2.30			0.21	0.82, 2.43
Home	2.4%	0.24*	0.16, 0.36			0.07	0.31, 1.05
**Skilled delivery provider**							
No	2.0%	1.0 (ref)				1.0 (ref)	
Yes	7.2%	3.83*	2.34, 6.26			0.78	0.56, 2.19

**Cesarean delivery** (n = 182)							
No	3.8%	1.0 (ref)				1.0 (ref)	
Yes	11.1%	3.14*	2.19, 4.51			0.25	0.82, 2.09
**Infant characteristics**							
**Low birthweight** (n = 145)							
No	8.9%	1.0 (ref)				1.0 (ref)	
Yes	2.2%	0.23*	0.07, 0.82			0.04*	0.07, 0.91

Notes: n are weighted counts, rounded to the nearest whole number. OR Odds Ratio, CI Confidence Interval, rounded to the nearest hundredth, Ref reference value.

* $p < 0.05$

†Geographic zones: Delta (Ayeyarwady, Bago, Yangon), Central (Magway, Mandalay, Naypyitaw, Sagaing), Coastal (Mon, Rakhine, Tanintharyi), Hilly (Chin, Kachin, Kayah, Kayin, Shan)

[b]Recommended ANC services included blood pressure, urine and blood tests from a skilled provider.

§Model 1 includes maternal sociodemographic characteristics (delivery year, age, education, household wealth, geographic zones, urban area).

¶Model 2 is adjusted for all variables in Model 1 as well as media, health system, and infant factors.
